# Caregiver strategies supporting community participation among children and youth with or at risk for disabilities: a mixed-methods study

**DOI:** 10.3389/fped.2024.1345755

**Published:** 2024-02-15

**Authors:** Vera C. Kaelin, Shivani Saluja, Dianna L. Bosak, Dana Anaby, Martha Werler, Mary A. Khetani

**Affiliations:** ^1^ Occupational Therapy, University of Illinois Chicago, Chicago, IL, United States; ^2^ Computer Science, University of Illinois Chicago, Chicago, IL, United States; ^3^ Children’s Participation in Environment Research Lab, University of Illinois Chicago, Chicago, IL, United States; ^4^ Computing Science, Umeå University, Umeå, Sweden; ^5^ School of Physical and Occupational Therapy, McGill University, Montreal, CA, United States; ^6^ CanChild Centre for Childhood Disability Research, McMaster University, Hamilton, CA, United States; ^7^ Epidemiology, Boston University, Boston, MA, United States

**Keywords:** attendance, involvement, pediatric rehabilitation, craniofacial microsomia, childhood-onset disability

## Abstract

**Introduction:**

The purpose of this mixed-methods study is to examine the role of caregiver strategies to support community participation among children and youth with disabilities and those at risk, from the caregiver perspective. For the quantitative phase, we tested the hypothesized positive effect of participation-focused caregiver strategies on the relationship(s) between participation-related constructs and community participation attendance and involvement. For the qualitative phase, we solicited caregiver perspectives to explain the quantitative findings.

**Methods:**

An explanatory sequential mixed-methods design (QUAN > qual) was used. For the quantitative phase, we conducted secondary analyses of data collected during a second follow-up phase of a longitudinal cohort study, including 260 families of children and youth (mean age: 13.5 years) with disabilities and those at risk [i.e., 120 families of children and youth with craniofacial microsomia (CFM); 140 families of children and youth with other types of childhood-onset disabilities]. Data were collected through the Participation and Environment Measure—Children and Youth, the Pediatric Quality of Life Inventory, and the Child Behavior Checklist and analyzed using structural equation modeling. For the qualitative phase, we conducted semi-structured interviews with eight caregivers of children and youth with disabilities and those at risk (i.e., three caregivers of children and youth with CFM; five caregivers of children and youth with other childhood-onset disabilities). Interviews were transcribed verbatim and inductively content-analyzed.

**Results:**

Our model reached acceptable to close model fit [CFI = 0.952; RMSEA = 0.068 (90% CI = 0.054–0.082); SRMR = 0.055; TLI = 0.936], revealing no significant effect of the number of participation-focused caregiver strategies on the relationships between participation-related constructs (e.g., activity competence, environment/context) and community participation in terms of attendance and involvement. The qualitative findings revealed three main categories for how caregivers explained these quantitative results: (1) caregiver workload and supports needed for implementing strategies; (2) caregivers careful strategy quality appraisal; and (3) community setting characteristics hindering successful strategy implementation.

**Discussion:**

The findings suggest that the insignificant effect of the number of caregiver strategies may be explained by the intensified need for caregiver effort and support to develop and implement quality strategies that are responsive to community setting characteristics.

## Introduction

Participation, defined as attendance and involvement in activities (1), is a key outcome of habilitation and rehabilitation (i.e., re/habilitation) services and an indicator of child and youth wellbeing in many settings, such as their home, school, and community (2). The family of Participation-Related Constructs (fPRC) (1) is a common framework in re/habilitation research based on evidence regarding salient predictors of child and youth participation (i.e., participation-related constructs including environment/context, child or youth activity competencies, preferences, and sense of self) to guide the design of participation-focused interventions for children and youth experiencing disability.

Caregivers use participation-focused strategies (e.g., offering support and guidance, planning for activities ahead of time, creating routines) when targeting predictor(s) to promote child and youth participation (3–6). Recent research indicates a significant positive effect of the number of participation-focused caregiver strategies on the relationship between school environmental supports and school participation attendance among children and youth with disabilities and those at risk (7). Similarly, a prior study of critically ill children revealed an effect of having participation-focused caregiver strategies on higher caregiver satisfaction with their child's home participation when combined with receiving pediatric re/habilitation services (8). Since these prior studies focused on home or school participation (7, 8), the role that caregiver strategies play in supporting child and youth community participation is less understood. The community setting becomes increasingly important as children age and transition into adulthood (9), yet it presents more barriers and a smaller variety of strategies (10, 11). Therefore, understanding how caregiver strategies support child and youth community participation is important for advancing participation-focused re/habilitation services.

Prior mixed-methods studies have advanced knowledge of participation disparities (12), guided the design of participation-focused re/habilitation interventions (13, 14), and identified their implementation parameters (15). To our knowledge, this is the first mixed-methods study to examine the specific role of caregiver strategies for supporting community participation among transition-aged children and youth with disabilities and those at risk (16). For the quantitative phase, we hypothesized a significant effect of the number of participation-focused caregiver strategies on the relationship(s) between participation-related constructs (behavior problems, physical functioning problems, community environmental features, and community environmental resources) and community participation attendance and involvement. For the qualitative phase, we aimed to solicit the perspective of caregivers of children and youth with disabilities and those at risk to explain the quantitative results. Specifically, our research question for the qualitative phase was “How do caregivers appraise their experience with participation-focused strategies within the community setting?”

## Materials and methods

### Study design

This study employed an explanatory sequential mixed-methods study design (QUAN > qual), with two distinct and sequential phases (i.e., follow-up explanations model): a quantitative phase followed by a qualitative phase to explain and elaborate on quantitative phase results (17, 18). This two-phase study, incorporating multiple data sources (i.e., triangulation), began with a cross-sectional analysis of secondary data that were collected as part of the second follow-up phase of a longitudinal cohort study of children and youth with and without craniofacial microsomia (CFM) (NIDCR R01 DE 11939; 2010–2015; PI: Dr. Werler) (i.e., quantitative phase) (19, 20). The second descriptive qualitative explanatory phase (21) included primary data collection with sampling from the population represented in the quantitative phase, as recommended for mixed-methods research involving secondary data (18). The original research project was approved by the institutional review board of Boston University and Seattle Children's Hospital prior to data collection and later approved at the University of Illinois Chicago for this study.

### Quantitative (QUAN) phase

#### Participants

The participants were families of children and youth with/at risk for disabilities [i.e., families of children and youth with CFM and children and youth with childhood-onset disabilities who receive health-related and/or educational services (e.g., occupational therapy, special education)] (22, 23) that were part of the second follow-up phase of a longitudinal cohort study. Originally, caregivers of children with CFM were included if their child (1) was younger than 36 months at the time of recruitment (i.e., during the first study phase of the longitudinal cohort study) and (2) was diagnosed with CFM by a physician, according to the established criteria for hemifacial microsomia, facial asymmetry, unilateral microtia, oculo-auriculo-vertebral syndrome, or Goldenhar syndrome (24, 25). Children and youth who were adopted or diagnosed with chromosomal anomalies, Mendelian-inherited disorders, or who were exposed to isotretinoin *in utero* were excluded from the study (24, 25). Families of children without CFM were included if their child (1) had no known birth defect, (2) was not adopted, and (3) was within 2 months of the age of children with CFM at the time of recruitment (24, 25). This resulted in a total cohort of 457 families, of which 302 were families of children and youth with/at risk for a disability (i.e., 142 families of children and youth with CFM and 160 families of children and youth with other childhood-onset disabilities who received health-related and/or educational services).

#### Data collection

This study involved secondary analyses of data that were collected between 2011 and 2015. Children and youth were tested by trained psychometrists who traveled to administer a 4–5 h battery of assessments in the child's natural environment (e.g., a private room at a library or local community center), while their caregivers completed proxy-reported questionnaires and received a $35 gift card (19, 20). The measure selection from the existing dataset was guided by the fPRC framework (1). Measures included the Participation and Environment Measure—Children and Youth (PEM-CY) (26) collecting data on community participation “attendance” and “involvement” and “participation-focused caregiver strategies” used in the community setting. The PEM-CY was also used to represent the participation-related construct “environment/context” by collecting data on “community environmental features” and “community environmental resources.” To represent the participation-related construct of “activity competence,” the Pediatric Quality of Life Inventory (PedsQL) 4.0 Parent-Proxy Report (PedsQL) (27) was used to measure “physical functioning problems,” and the Child Behavior Checklist (CBCL) (28) was used to measure “behavioral problems.” We created two latent variables for participation (i.e., community participation attendance, community participation involvement), which serve as endogenous variables in the tested model. In addition, we created five latent variables, which serve as exogenous variables in the tested model. Those include one latent variable for participation-focused caregiver strategies, two latent variables measuring environmental constructs (i.e., community environmental features, community environmental resources), and two latent variables measuring the activity competence construct (i.e., physical functioning problems, behavioral problems). Child age and caregiver education were included as confounding variables. Further information regarding the created latent variables and the included measures is summarized in Table 1.

**Table 1:** Latent variables and used measures.

Latent variable	Measure	Measure's Psychometrics
Participation Constructs		
Community Participation Attendance	This latent variable was created using 10 items or activity sets of the PEM-CY community section, which asks caregivers about their child's or youth's frequency of participation (from never = 0 to daily = 7) in 10 types of community activities (e.g., social gatherings and community events).	The PEM-CY participation frequency scale for the community participation section has good test–retest reliability (ICC = 0.79) and internal consistency (*α *=* *0.70) for large sample research (29), with a similar Cronbach's alpha estimated for this study (*α *=* *0.67) (22). The latent variable for community participation attendance (frequency) was previously confirmed (30).
Community Participation Involvement	This latent variable was created using 10 items or activity sets of the PEM-CY community section, which asks caregivers about their child's or youth's level of involvement (from not very involved = 1 to very involved = 5) in 10 types of community activities.	The PEM-CY participation involvement scale for the community participation section has acceptable to good test–retest reliability (ICC = 0.69) and internal consistency (*α *=* *0.79) for large sample research (29), with a similar Cronbach's alpha estimated for this study (*α *=* *0.84) (22). The latent variable for community participation involvement was previously confirmed (30).
Participation-Focused Caregiver Strategy Construct		
Participation-Focused Caregiver Strategies	The single-indicator latent variable for the number of disclosed “participation-focused caregiver strategies” used in the community setting was created with data collected with open-ended PEM-CY items. Caregivers administrating the PEM-CY community section were asked to describe up to three strategies used to support their child's participation in community activities, yielding up to 780 strategies. To exclude entries that do not qualify as strategy (e.g., responses such as “N/A”), narrative data on caregiver strategies were first screened by two research staff (JS, ZS). A decimal score (0.0, 0.33, 0.67, 1.0) was calculated by dividing the number of provided caregiver strategies by the maximum number of strategies possible (i.e., 3).	This single-indicator latent variable was employed in a previous study that applied structural equation modeling to examine its effect on the relationships between participation-related constructs and participation attendance and involvement (7).
Environmental Constructs		
Community Environmental Features	The latent variable for “community environmental features’ was created using 9 items of the PEM-CY community section asking caregivers about environmental features (e.g., physical layout) that support or hinder their child's or youth's participation (from usually makes it harder/usually no = 1 to usually helps/usually yes/no impact = 3).	The PEM-CY community environmental section has good test–retest reliability (ICC = 0.96) and internal consistency (*α *≥* *0.80) for large sample research (29), with a similar Cronbach's alpha estimated for this study (*α *=* *0.81) (22) and also for community environmental features specifically (*α *=* *0.79).
Community Environmental Resources	The latent variable for “community environmental resources” was created using 7 items of the PEM-CY community section asking caregivers about environmental resources (e.g., money) that support or hinder their child's or youth's participation (from usually makes it harder/usually no = 1 to usually helps/usually yes/no impact = 3).	The PEM-CY community environmental section has good test–retest reliability (ICC = 0.96) and internal consistency (*α *≥* *0.80) for large sample research (29), with a similar Cronbach's alpha estimated for this study (*α *=* *0.81) (22) and also for community environmental resources specifically (*α *=* *0.80).
Activity Competence Constructs		
Physical Functioning Problems	This latent variable was created using eight items of the physical functioning subscale of the PedsQL (27), which asks caregivers about the level of problems their child had in the past months with physical functions such as running or their energy level (from never = 0 to almost always = 4).	The PedsQL 4.0 has evidence of internal consistency reliability ranges from *α *=* *0.85–0.89 for the physical functioning scale and content validity by replicating known-group differences (27).
Behavioral problems	This latent variable was created using all 67 items of the CBCL (28), which asks caregivers about the frequency of behavioral problems in children and youth among 32 items on caregiver observation of their child's externalized behavior problems (e.g., hyperactive, disruptive) and 35 items on observed internalizing problems (e.g., withdrawn, despondent).	The CBCL has evidence of one week test–retest reliability (ICC = 0.95), internal consistency reliability (*α *=* *0.78–0.97) and content validity by replicating known-group differences (28).
Confounder variables		
Child age	This single-indicator latent variable was created using one item of the demographic questionnaire.	“Child age” was chosen due to its strong association with participation (30).
Caregiver education	This single-indicator latent variable was created using one item of the demographic questionnaire.	“Caregiver education” was chosen due to its strong association with participation (31). We chose “at least high school/general education diploma” as the reference group for caregiver education.

PEM-CY, participation and environment measure for children and youth; ICC, intraclass correlation coefficient; PedsQL, pediatric quality of life inventory; CBCL, child behavior checklist.

#### Data analyses

We used SAS 9.4 (32) to conduct descriptive statistics and bivariate correlations. We excluded participants (*n* = 42) with missing data on all participation variables, resulting in 260 participants with data on variables of interest for this study. Demographic characteristics among excluded and included families differed significantly in their distribution of caregiver educational background (*χ*^2^ = 19.89; *p* < 0.05), total annual income (*χ*^2^ = 42.04; *p* < 0.05), and child/youth race and ethnicity (*χ*^2^ = 68.14; *p* < 0.05). The included families were more likely to have a higher proportion of caregivers who had earned higher levels of education and annual income and children or youth of White non-Latinx race/ethnicity.

The main analyses were conducted using MPlus version 7 software (33). We applied structural equation modeling (SEM) to test the structural models while accounting for the construct of the latent variables using a fixed-factor method. To identify an optimal parceling scheme to represent the latent variables for these analyses, we conducted item-level confirmatory factor analyses (CFAs) as supplemental analyses (34). The decimal score of caregiver strategies and the covariates (i.e., child age and caregiver education) were included in the models as single-indicator latent constructs using the fixed-factor method and setting the residual variance to zero.

To test the effect of the number of participation-focused caregiver strategies in explaining the relationship between the exogenous variables for participation-related constructs (i.e., physical functioning problems, behavioral problems, community environmental features, and community environmental resources) and the endogenous variables for community participation (i.e., attendance and involvement), we used the MODEL INDRIECT command in MPlus version 7, with bias-corrected bootstrap resampling (5,000 samples), to improve the accuracy of the standard error estimates (35). After fitting a saturated model (i.e., all potential paths included), we removed non-significant paths one-by-one, provided that there was no significant decrease in model fit as measured by Chi-squared difference testing. This resulted in a pruned and final model.

We evaluated model fit through the Comparative Fit Index (CFI), the Root Mean Square Error of Approximation (RMSEA), the Standardized Root Mean Squared Residual (SRMR), and the Tucker–Lewis Index (TLI). The acceptable fit indices are ≥.90 for CFI and TLI and ≤0.08 for RMSEA and SRMR (36). Chi-square values were reported but not used to evaluate model fit (36, 37).

### Qualitative (QUAL) phase

#### Participants

Caregivers of children and youth with/at risk for disabilities, representing the same population as the quantitative phase (i.e., children and youth with CFM and children and youth with a childhood-onset disability), were recruited between 2022 and 2023 through a US-based non-profit organization and via snowball sampling. The eligible participants met the following inclusion criteria: (1) they identified as the parent/legal guardian of a child 11–17 years old who receives health-related and/or educational services and/or is diagnosed with CFM; (2) they can read, write, and speak English; and (3) they have internet access.

#### Data collection

The research staff sent an email to eligible and interested caregivers with a REDCap (38, 39) link to (1) confirm the eligibility of the caregivers for the study; (2) provide their informed consent; (3) complete a demographic questionnaire; and (4) provide interview availability. Individual semi-structured Zoom interviews (35–60 min) were co-facilitated by two authors (SS, VK) to further explain the main quantitative results. VK had prior experience with qualitative and mixed-methods research. During the semi-structured Zoom interviews, the participants were asked to interpret the non-significant effect of the number of participation-focused caregiver strategies in the presence of other important factors (i.e., child and youth activity competencies, environmental supports) (see Supplementary Data Sheet). This was done in part by discussing the qualities of strategies represented in the quantitative dataset, targeting different participation-related constructs (e.g., strategies targeting the environment: “Try to determine what is happening well ahead of time and plan for it”; strategies targeting a child's or youth's sense of self: “We always offer words of encouragement and praise”; see Supplementary Data Sheet) to further explain these main quantitative results. The interviews were recorded and transcribed verbatim. Caregiver recruitment continued until additional data no longer yielded significant new information pertinent to the research question, signifying the attainment of data saturation. The participants were issued $30 electronic gift cards.

#### Data analysis

The qualitative data analysis was guided by the Rigorous and Accelerated Data Reduction (RADaR) technique (40), while qualitative content analyses were performed using the approach developed by Elo and Kyngäs (41). Data were first organized into a five-column table, which included the (1) transcript number, (2) question number, (3) participant's response, (4) code, and (5) notes (40). Two authors (SS, VK) carefully examined participant responses (i.e., transcripts) to select the data relevant to the research aim. These relevant data were then organized in a new (i.e., reduced) five-column data table (40) and independently analyzed by the same two authors (SS, VK) using inductive content analyses (41). First, initial open coding was conducted by reading and re-reading the participant responses line-by-line and adding notes next to the text using the “code” and “notes” columns. In the “code” column, we entered notes representing “condensed meaning units,” and in the “notes” column, we added further thoughts and comments to support the analysis process. Coding discrepancies were discussed during regular meetings, and the text with similar “condensed meaning units” were grouped into sub-categories. In an iterative coding process, sub-categories were added, adjusted, and compared to ensure they were distinct. In the final abstraction phase, we collapsed sub-categories into categories and then further grouped these categories into three main categories (41). A preliminary summary of both quantitative and qualitative results was shared with a research advisory board to help finalize and interpret the results.

## Results

### Quantitative phase (QUAN) results

#### Sample characteristics

The participants were 260 families of children and youth with/at risk for disabilities (i.e., 120 children and youth with CFM; 140 children and youth with other types of childhood-onset disabilities who receive health-related and/or educational services). Most families were White and non-Latinx and had an annual income of at least $65,000. Families had a relatively diverse educational background. The age range of the children and youth is between 11 years, 1 month and 17 years, 5 months. Most children and youth received at least one type of educational and/or health-related service (Table 2).

**Table 2:** Child, youth, and family characteristics and service use.

Characteristics and service use	*N* = 260
Child/youth gender (male), *n* (%)	143 (55.00)
Child/youth age, *M* (*SD*)	13 years 6 months (1 years 5 months)
Child/youth race/ethnicity, *n* (%)	
White, non-Latinx	215 (82.69)
White, Latinx	24 (9.23)
African American	8 (3.08)
Other	13 (5.00)
Receiving at least one type of health-related and/or educational service, *n* (%)^a^	234 (90.35)
Type of service received, *n* (%)^a^	
Re/habilitation services (OT, PT, ST)	150 (58.82)
Vision therapy	33 (12.74)
Hearing services	42 (16.28)
Mental health services	54 (20.85)
Special education services	81 (31.64)
Other services	93 (36.19)
Caregiver education, *n* (%)^a^	
At least high school/GED	73 (29.08)
Associates degree	42 (16.73)
Bachelor’s degree	85 (33.86)
Graduate degree	51 (20.32)
Caregiver annual income, *n* (%)^a^	
<$25,000	21 (8.57)
$25,000–$34,999	25 (10.20)
$35,000–$64,999	35 (14.29)
≥$65,000	164 (66.94)

OT, occupational therapy; PT, physical therapy; ST, speech/language therapy; GED, general education diploma.

^a^ Missing data.

#### Structural model

We applied item-level CFA to create 15 parcels for latent constructs (Supplementary Image). Three latent constructs had two indicators (i.e., community participation attendance, community environmental features, behavioral problems), and three latent constructs had two indicators (i.e., community participation involvement, community environmental resources, physical functioning problems), with standardized loadings ranging from 0.55 to 1.0. Per modification index, we allowed for residuals of two parcels to correlate (i.e., residuals of parcel 2 of the behavioral problem with residuals of parcel 3 of the physical functioning problems construct). Our model reached acceptable close model fit (CFI = 0.952; RMSEA = 0.068 [90% CI = 0.054–0.082]; SRMR = 0.055; TLI = 0.936), despite significant *χ*^2^ testing (*χ*^2^(78) = 171.151; *p* < 0.05).

#### Effects of participation-focused strategies

**Figure 1:**
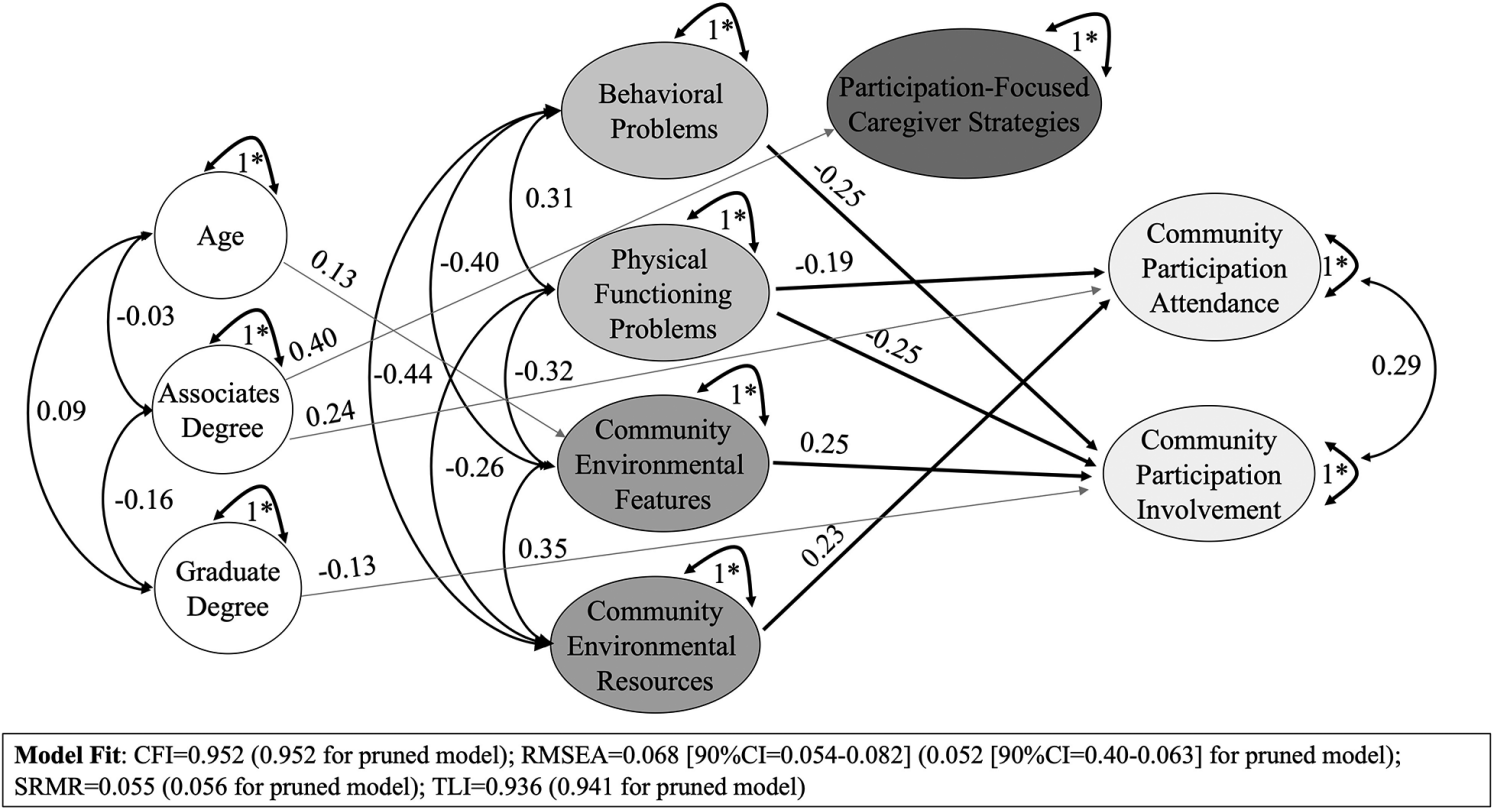
Effects of participation-focused caregiver strategies on relationships between predictors and community participation attendance and involvement.

There were no significant effects of the number of disclosed participation-focused caregiver strategies found on any relationship between predictors (activity competence, environmental factors) and community participation (attendance or involvement) (Figure 1).

### Qualitative phase (QUAL) results

#### Sample characteristics

The participants were eight caregivers of children and youth with disabilities and those at risk (i.e., three caregivers of children and youth with CFM; five caregivers of children and youth with other types of childhood-onset disabilities who receive health-related and/or educational services; see Table 3). The participating caregivers had relatively diverse levels of educational attainment, and half of the sample had an annual family income below the US median income level (i.e., $70,800) (42). Most caregivers were White and non-Latinx. Children and youth were between 11 years, 6 months and 16 years, 8 months old and received at least one type of health-related and/or educational service.

**Table 3:** Child, youth, and family characteristics (62).

Participant	Child/youth gender	Child/youth age	Child/youth diagnosis	Child/youth race/ethnicity	Caregiver race/ethnicity	Caregiver's formal education	Annual income
R1	Male	15 years, 8 months	CFM (unilateral facial asymmetry)	Black or African American	Black or African American	Missing information	$40,001–$50,000
R2	Male	12 years, 3 months	CFM (Goldenhar Syndrome/facial asymmetry)	American Indian/Alaskan Native	American Indian/Alaskan Native	Bachelor’s degree	$40,001–$50,000
R3	Female	13 years	CFM (Grade III, Unilateral Microtia and Aural Atresia of the right ear, slight craniofacial microsomia)	White	White	Bachelor’s degree	More than $100,000
R4	Female	16 years, 8 months	Autism spectrum disorder	White	White	Bachelor’s degree	More than $100,000
R5	Male	13 years, 1 month	Autism spectrum disorder and ADHD	White	White	Bachelor’s degree	More than $100,000
R6	Male	12 years	Down syndrome/Trisomy 21	White	White	Graduate degree	More than $100,000
R7	Male	11 years, 6 months	Autism spectrum disorder	White	White	High school graduate; diploma or equivalent (e.g., GED)	$50,001–60,000
R8	Male	14 years, 9 months	Autism spectrum disorder	Black or African American	Black or African American	Associates degree	$40,001–50,000

GED, general education diploma; CFM, craniofacial microsomia; ADHD, attention-deficit/hyperactivity disorder.

### Caregivers explaining the non-significant role of caregiver strategies to support community participation

**Figure 2:**
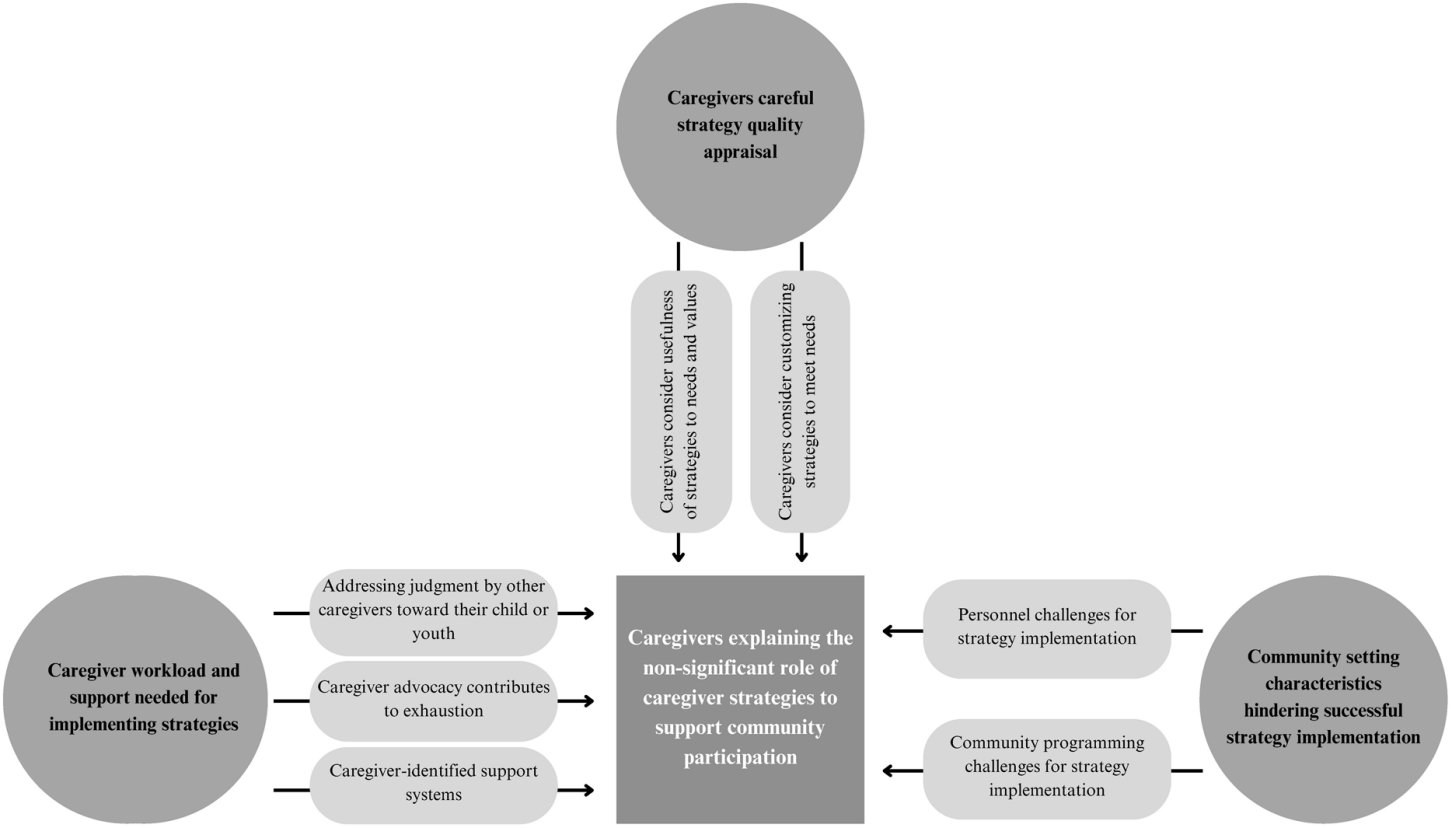
Caregiver explanation of non-significant role of caregiver strategies to support community participation.

The qualitative analyses revealed three main categories related to caregivers explaining the quantitative results: (1) the caregiver workload and the support needed for implementing strategies; (2) caregivers careful strategy quality appraisal; and (3) the community setting characteristics hindering successful strategy implementation (Figure 2).

#### Caregiver workload and support needed for implementing strategies

Three categories have been identified in relation to the workload and support that caregivers encounter, and how this can affect their implementation of strategies to support community participation: (1) addressing judgment by other caregivers toward their child or youth; (2) caregiver advocacy contributing to exhaustion, and (3) caregiver-identified support systems.

##### Addressing judgment by other caregivers toward their child or youth

Four out of eight caregivers shared their frustration related to community members judging their child or youth with a disability or at risk for a disability, resulting in additional challenges for caregivers to support their child or youth's participation in the community setting. This included disrespect from other caregivers of children who have not experienced disabilities. R7 shared, “*I feel like there is some judgment, you know, or like […] not from parents that have experienced it, but just […] you feel like they’re kind of like, how can your child be this way.*” Caregivers described their extra work in dealing with these encounters. R3 described:

*There was one time where I was almost beside myself. It was it was heartbreaking. […] There was a mom sitting next to me with her daughter and her daughter was just staring at my daughter at her ear. And actually asked her mother loud enough to where I could hear her say, What's wrong with that girl? What's wrong with her ear? […] And the mother just ignored her daughter. […] She didn't say anything. […] [My daughter] wasn't bothered by it, thank God. But I was. […] I had to deal with that on the side*.

##### Caregiver advocacy contributes to exhaustion

Six out of eight caregivers described advocacy work related to sharing knowledge to make community programs more inclusive, educating other families, children, and youth regarding disabilities, and helping other families of children or youth with/at risk for disabilities. Caregivers mentioned how this additional work is important but exhausting as it is in addition to similar work they undertake in the school setting: “*It's a lot for the parent*” (R7) and “*I am tired of, you know, because I do try to advocate and I try, you know, there's many things*” (R4). Some caregivers described prioritizing their advocacy efforts to promote school participation and decided to take a break from implementing strategies when their child attended community activities. R3 describes, “*I'm invested in our teachers and our school for my daughter to be educated. And it's very important she gets the basics out of life. When she goes to a sport, it's fun time. […] So it's kind of an outlet for the parents. It's an outlet for the kids.*”

##### Caregiver-identified support systems

Six out of eight caregivers shared examples of support systems when implementing strategies and managing their workload to facilitate their child's or youth's community participation. This included sharing their workload with family members, receiving support from friends and families in similar situations, and seeking help from professionals and non-governmental organizations (NGOs). For example, R2 mentioned, “*My son is there to help,*” and R4 shared, “*It's been my husband and I tag-teaming.*” Some caregivers (*n* = 4) shared how they reach out to friends to experience support. R3 shared, “*We have lots of friends that are military families and […] my circle of mom friends, we all talk about, like, how things are going at home and what we're happy with or not happy with, and the struggle of raising your kids as they go through the different ages and how you as a parent are choosing to address it. It's a struggle, you know, so we kind of learn from each other.*”

Five out of eight caregivers shared how professionals and organizations provide assistance, such as through therapy for caregivers, support groups, and collaboration with their public schools to help implement participation-focused strategies in their community. For example, when asked what they would advise a novice parent, R3 replied, “*Consider therapy. Therapy is not a negative thing. You know, I used to never believe in support groups and all that, and now I run five of them*” and R5 shared, “*So, [name NGO] has been a wonderful support. We went there when he was a newborn and […] now I'm kind of finding my way back to them*.”

#### Caregivers careful strategy quality appraisal

Two categories emerged related to how caregivers appraise the quality of the strategies used to support their child's participation in the community: (1) caregivers consider the usefulness of participation-focused strategies relative to their current needs and values, and (2) caregivers consider customizing participation-focused strategies to meet their current needs.

##### Caregivers consider usefulness of strategies to needs and values

Seven out of eight caregivers confirmed the usefulness of participation-focused caregiver strategies from the dataset and as shared with them during the interviews. Caregivers described positive experiences with applying a participation-focused strategy, such as R7 who shared, “*It's actually my number one strategy all the time,*” and R6 sharing, “*[Child's name] is very much a schedule kid and likes to know the steps. […] So, I totally agree with that [strategy] about letting the kiddo know ahead of time, especially for a new activity or something unfamiliar. I think that's very important.*”

Caregivers appraised the usefulness of strategies shared with them relative to their current needs and values. Some caregivers perceive a particular participation-focused strategy as useful for their current situation, while other caregivers mentioned less success or less interest in using that same strategy for their current situation. For example, R7 explained that the strategy of taking “…*Favorite snacks to community events. I think that's an important one,*” whereas R5 shared: “*The snacks are maybe like down the list a little bit, but I have used those to have [my kid] stay on task and follow the plan during activity […] But I'm trying to kind of pull back on those, like, food rewards.*” Similarly, while R8 perceived the strategy of “We tell him how he can help others” as less useful to implement “*because […], he's not really a fan of people that much,*” R6 was inspired to use it more often: “*I need to do more of that. How he's helping other people.*”

##### Caregivers consider customizing strategies to meet needs

All caregivers (*n* = 8) shared similar or adapted strategies that they have used to support their child's or youth's community participation. This included examples that incorporated parts of a presented strategy or examples of different strategies using a similar approach. For instance, R2 expanded on the strategy of “taking favorite snacks to community events” by sharing, “*Maybe you wanna borrow something and somebody's like no, these are mine. I can’t give you, I can’t give this to you. So just get your belongings and, and you'll be OK.*”

Some caregivers (*n* = 4) also shared additional strategies. For example, R5 shared how they include their child or youth in decision-making about family vacations: “*[We] let him help plan our vacations and, you know, have a discussion on that so that we plan for it a little bit*,” and R3 shared how they find a friend to participate with their child: “*We find a friend. A friend that will do it with us.*”

#### Community setting characteristics hindering successful strategy implementation

Two categories emerged related to community setting characteristics that hinder the successful implementation of participation-focused caregiver strategies: (1) personnel challenges for strategy implementation and (2) community programming challenges for strategy implementation.

##### Personnel challenges for strategy implementation

All caregivers (*n* = 8) perceived personnel knowledge, experience, effort, and interest as influencing their customized implementation of participation-focused caregiver strategies in the community. For example, R3 shares: “*It's really up to that coach or that person who's heading up that team. Their approach, their technique, some are better than others*. Most caregivers (*n* = 5) noted a lack of knowledge on disability and inclusion among community personnel relative to school personnel. For example, R3 shared, “*The coach is not going to be this IEP teacher who went to school with the passion to help a child who's deaf and hard of hearing. They may have no background on hearing loss*.”

Five out of eight caregivers described the impact of limited lack of structure and consistency within the community setting (e.g., lack of routines, including a high turnover among staff) to support participation. R8 described, “*[In] the community, people [staff] kind of come and go, you know, they don't really take it seriously […]. If you hire [community staff], let's say for like a week or two weeks, they [are] really not going to put in the time and energy because they know they're not going to be there long*.” Caregivers (*n* = 4) described how this lack of structure and consistency hindered their communication with community staff as well as community staff's familiarity with their child or youth, which, in turn, hindered their ability to implement strategies to support their child or youth's participation in community activities. R3, for example, shared “*I think […] it comes down to communication. So, for example, with my daughter's IEP, we absolutely communicate with her teachers all the time. […] This helps tremendously with carrying [successful strategies].*” Similarly, R7 shared: “*[At] T-ball, they're not going to know [that] my child has a huge fear of dogs. [If] there was somebody that brought their dog […] they wouldn't really know […] oh, he's afraid of dogs, […] that's why he's acting this way and trying to, you know, use strategies for that*.”

##### Community programming challenges for strategy implementation

Four out of eight caregivers experienced a lack of diversity and inclusion in community activities. They noted that their children and youth were often the only ones with disabilities or at risk for a disability attending a community event, which discouraged caregivers from implementing strategies to support their child's or youth's participation. R1 explained “*So you know in a community setting, […] I like my kid interacting with other kids who have the same condition to his. [..] So maybe two to three kids who can share some knowledge with him.*” This lack of diversity specifically in the community setting was also observed by R2: “*[In] the community setting […] you see nobody with, with your [child's] condition. So like you are just alone and you…so you feel like discouraged*.” Caregivers described the need for more inclusive community programs, such as when R4 shared, *“[We] took her to see the Lion King sensory friendly show […]. Um, and it was fabulous. […] it felt nice to feel included. It's like how we felt when we went to Disney to be able to be respected and supported and have those supports there if we needed them.*” However, inclusive programming was seen as rare, as R7 shared, “*Community stuff, like it's either like offered for like children that are more like severely have needs and then it's just for like average children […] and then […] you don’t have anything really in the middle, you know.*”

## Discussion

This mixed-methods study extends knowledge about the role of caregiver strategies to support child and youth community participation. The results from the quantitative phase revealed no significant effect of the number of participation-focused caregiver strategies on the relationships between participation-related constructs (i.e., community environmental features and supports, child physical functioning, and behavioral problems) and community participation (attendance and involvement). This result partly contrasts the findings of prior research indicating that having a greater number of strategies can intensify the positive impact of environmental support on participation (7). The reasons for this discrepancy might be related to our qualitative results explaining our non-significant result, revealing setting-specific challenges that caregivers experience, their additional workload, and careful appraisal of strategy quality when trying to implement strategies in the community.

Caregivers observed multiple community setting characteristics related to personnel and programming that may hinder the successful implementation of participation-focused caregiver strategies (e.g., personnel's limited knowledge on disability and inclusion and lack of consistency, structure, and diversity) and, thus, may help explain the non-significant effect of participation-focused caregiver strategies on relationships between participation-related constructs and community participation. Caregivers in our study noted how these challenging characteristics differed from those in the school setting, potentially reflecting the influence of policies and regulations on the structure, inclusiveness, and support available in a certain setting. For example, schools are required to have Individual Education Program (IEP) meetings (43), which can support the implementation of participation-focused caregiver strategies. Similar requirements for the community setting are missing and therefore may have contributed to caregiver exhaustion, as reinforced by prior research indicating additional mental and physical workload across settings (i.e., school, community) among caregivers of children and youth with or at risk for disabilities (44, 45). Caregivers valued their personal and professional support systems to manage exhaustion while prioritizing their investment in strategies to support school participation rather than community participation. School participation may be prioritized because it is perceived to be more supported by professionals and important for their child's or youth's basic for life. If caregivers assign less importance to community participation, this perspective could contribute to explaining the quantitative findings indicating an insignificant positive effect of caregiver strategies on the relationships between participation-related constructs (i.e., activity competencies, environmental factors) and community participation (i.e., attendance and involvement). In other words, caregivers may opt to allocate their resources toward implementing participation-focused strategies in the school setting rather than the community setting. This prioritization could potentially lead to the impact of participation-focused caregiver strategies in the school setting compared with the community setting. Alternatively, our results may indicate that caregivers temper their expectations to support participation attendance (vs. seeking to support both attendance and involvement), potentially further explaining current and prior quantitative findings about a lack of positive effect of participation-focused caregiver strategies on relationships related to involvement (7). This finding has prompted research that is underway to characterize a stepwise process for how caregivers might create strategies to support community participation.

Interventions such as the Pathways and Resources for Engagement and Participation (PREP) (46–50) and programs such as the Local Environment Model (LEM) (51, 52), where re/habilitation professionals work with stakeholders such as community personnel directly to implement participation-focused strategies, might be one way to reduce workload concerns among caregivers trying to support community participation. This collaboration may also enhance the community personnel's knowledge of disability and inclusion, thus supporting efforts toward a more inclusive community environment. Approaches such as the PREP and LEM require re/habilitation professionals to move from hands-on therapy to coaching (50, 53). Whereas hands-on therapy is therapist-led, coaching represents a family-led intervention where children, youths, and their families are encouraged to propose solution-focused strategies to overcome participation barriers. For example, within the context of PREP, individuals such as caregivers, children or youth, and community personnel are coached by a re/habilitation professional using coaching principles (54) (e.g., setting a participation goal for a self-chosen activity, guiding individuals in reflecting about barriers to participation) on modifying the environment to enhance activity accessibility and inclusion (47, 50). Interestingly, our data collection approach of exposing caregivers to existing participation-focused strategies encouraged them to share their own strategies or come up with strategies they would like to try in the future. This finding may support prior research revealing positive caregiver feedback regarding a strategy exchange feature within the Participation and Environment Measure Plus (PEM+) intervention, which facilitates sharing participation-focused strategies among caregivers when developing a re/habilitation care plan for their child (55).

Caregivers in this study were skilled in appraising strategy quality according to how useful and customizable they are relative to the families' current needs. This finding aligns with prior research emphasizing the importance of the context when supporting child and youth participation (1, 46, 50, 56). The disclosed caregiver strategies in our dataset were context-specific, based on the way the strategies were collected for a specific setting [i.e., “What are some things that you or other family members do that help your child participate successfully in activities in the community?” (26)]. However, our results may reinforce the need for future studies to capture greater specificity in the type of strategy, either by examining strategies specific to types of school or community activities when possible by the PEM version (57) or by the type of caregiver strategy reported (e.g., whether the strategy targets the child's environment/context, activity competencies, sense of self, or preferences) as can be classified (5, 58).^1^ These approaches may help to strengthen modeling of their effect on participation attendance and involvement, pending access to larger sums of data despite frequent recruitment issues in a re/habilitation population (59, 60).

### Limitations

This study is subject to several limitations. First, we were limited to existing data on select demographic and clinical characteristics, which may have limited sample description and confounder selection in the quantitative phase. Second, our sample was relatively diverse with respect to caregiver education; however, the included caregivers for the quantitative phase were more likely to have a higher educational background and income, and children's and youth's races and ethnicities were more likely White and non-Hispanic when compared with the participants we excluded due to missing data on all participation variables of interest. For our qualitative phase, our sample was more diverse in terms of annual income and race/ethnicity. However, snowball sampling may have led to a higher representation of more severe cases in the qualitative part compared with the quantitative part. Increasing sample diversity and reducing snowball sampling in future studies might be supported by efforts to create registries, such as for people with CFM (61). Third, we may have misclassified missing data when deriving a score for participation-focused caregiver strategies, as we cannot ascertain why 55% of caregivers did not report all three strategies. Fourth, the data on child or youth diagnosis were collected by physician reports in the quantitative phase and by caregiver reports in the qualitative phase, resulting in limited data on condition severity or diagnostic characteristics. Future research may benefit from using more detailed checklists to describe child or youth diagnostic characteristics.

## Conclusion

This mixed-methods study sought to examine the role of caregiver strategies for supporting community participation among transition-aged children and youth with disabilities and those at risk. Our quantitative findings indicated no significant effect of participation-focused caregiver strategies on the relationships between participation-related constructs and community participation. These results contradict the findings of prior research on school participation and can be partly explained by our qualitative results revealing differences in community setting characteristics (e.g., additional caregiver workload demands to implement strategies in the community setting, lack of community personnel's knowledge on disability and inclusion, and lack of consistency, structure, diversity, and inclusion in community activities) that may result in additional challenges when implementing strategies to promote participation attendance and involvement in this setting. Our findings emphasize the importance of targeting the community setting when developing and implementing strategies that prioritize caregiver participation.

## Data Availability

The datasets for this article are not publicly available due to concerns regarding participant/patient anonymity. Requests to access the datasets should be directed to the corresponding author.

## Footnotes

^1^Valizadeh M, Kaelin V, Khetani M, Parde N. CareCorpus: A corpus of real-world solution-focused caregiver strategies for personalized pediatric rehabilitation service design. (under review).
