# Supplementary figures and images for: Caregiver strategies supporting community participation among children and youth with or at risk for disabilities: a mixed-methods study
Source: Front Pediatr. 2024 Feb 15;12:1345755. doi: 10.3389/fped.2024.1345755 (PMC10902462; doi:10.3389/fped.2024.1345755)

# Confirmatory Factor Analysis

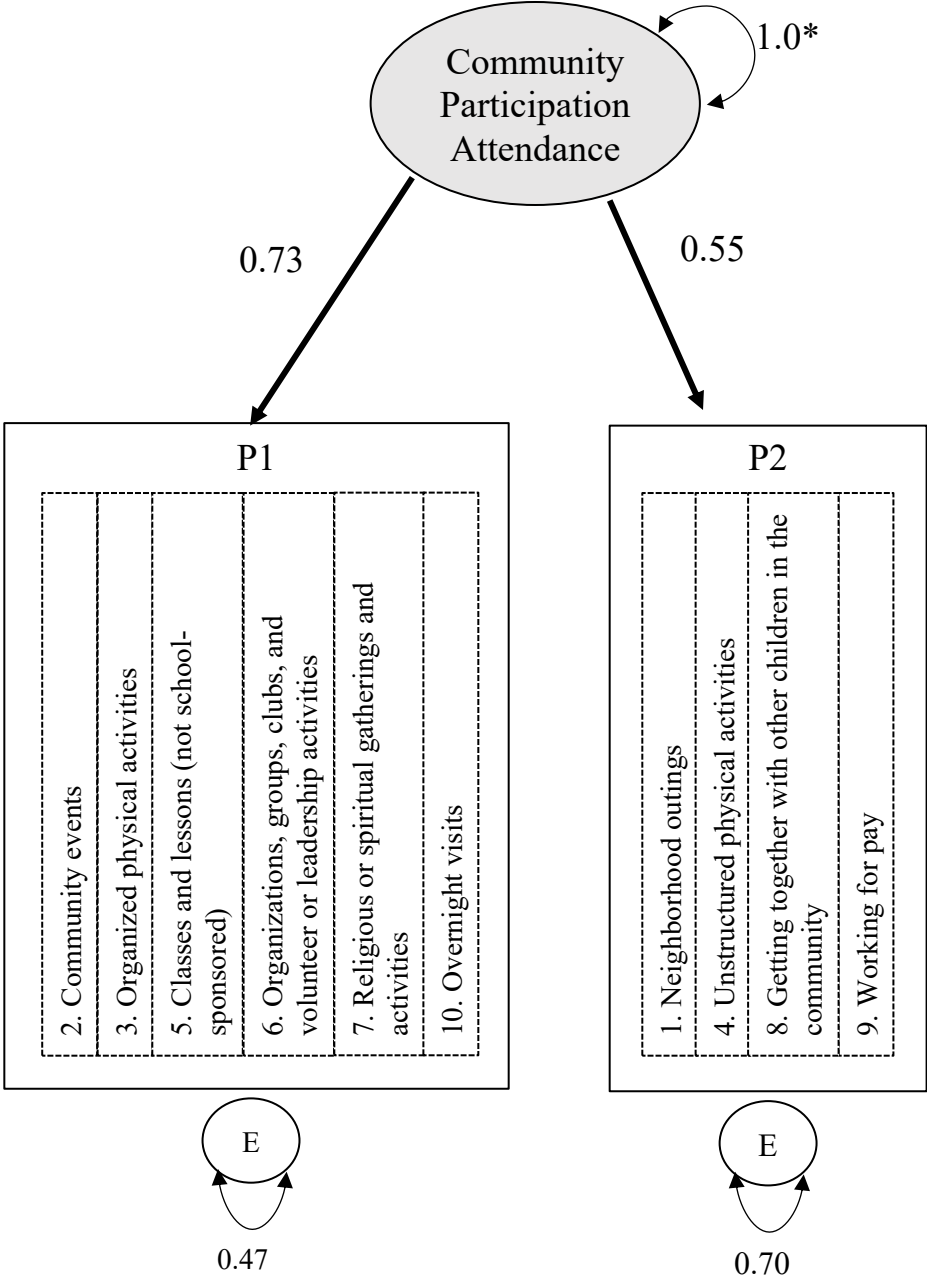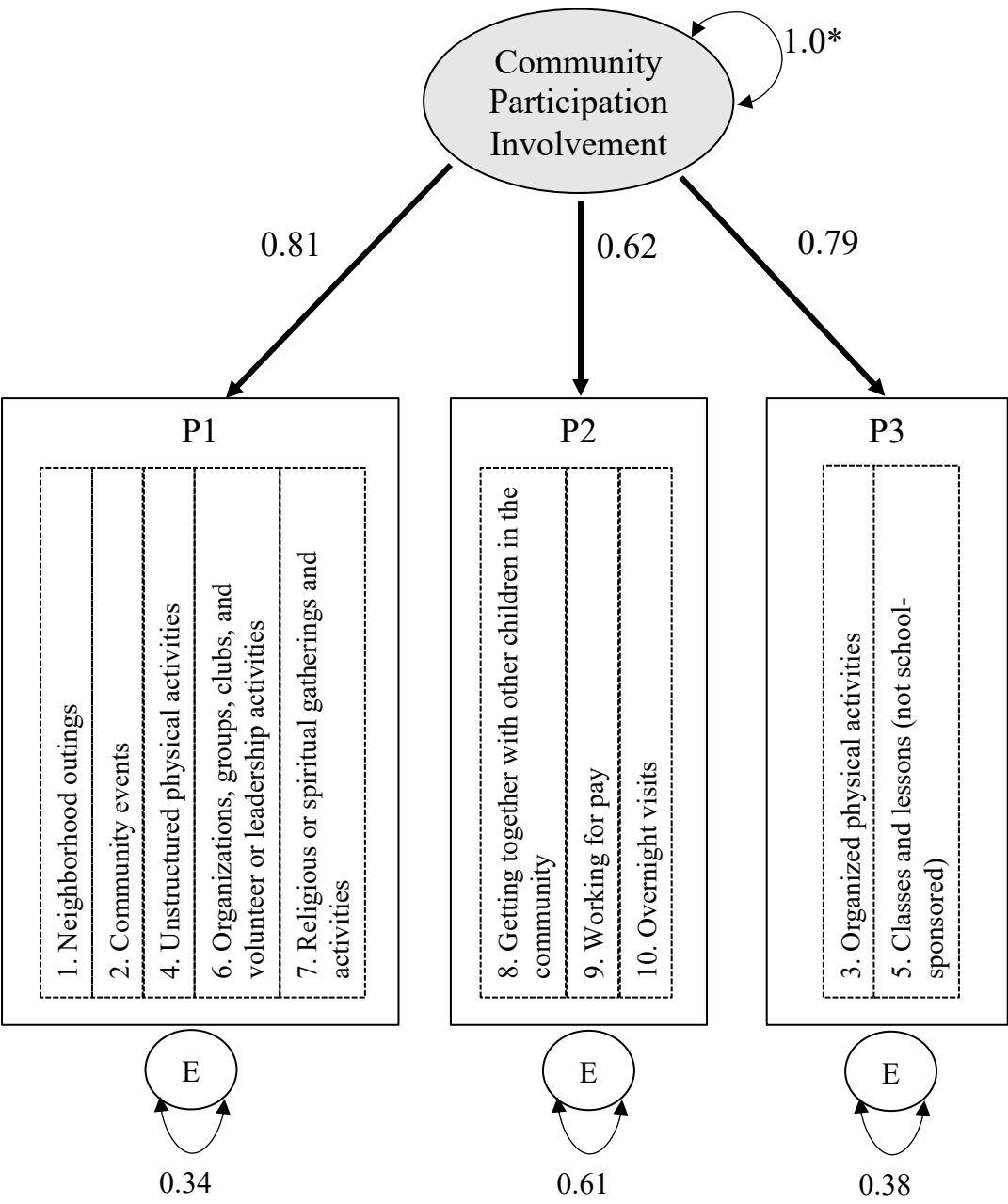

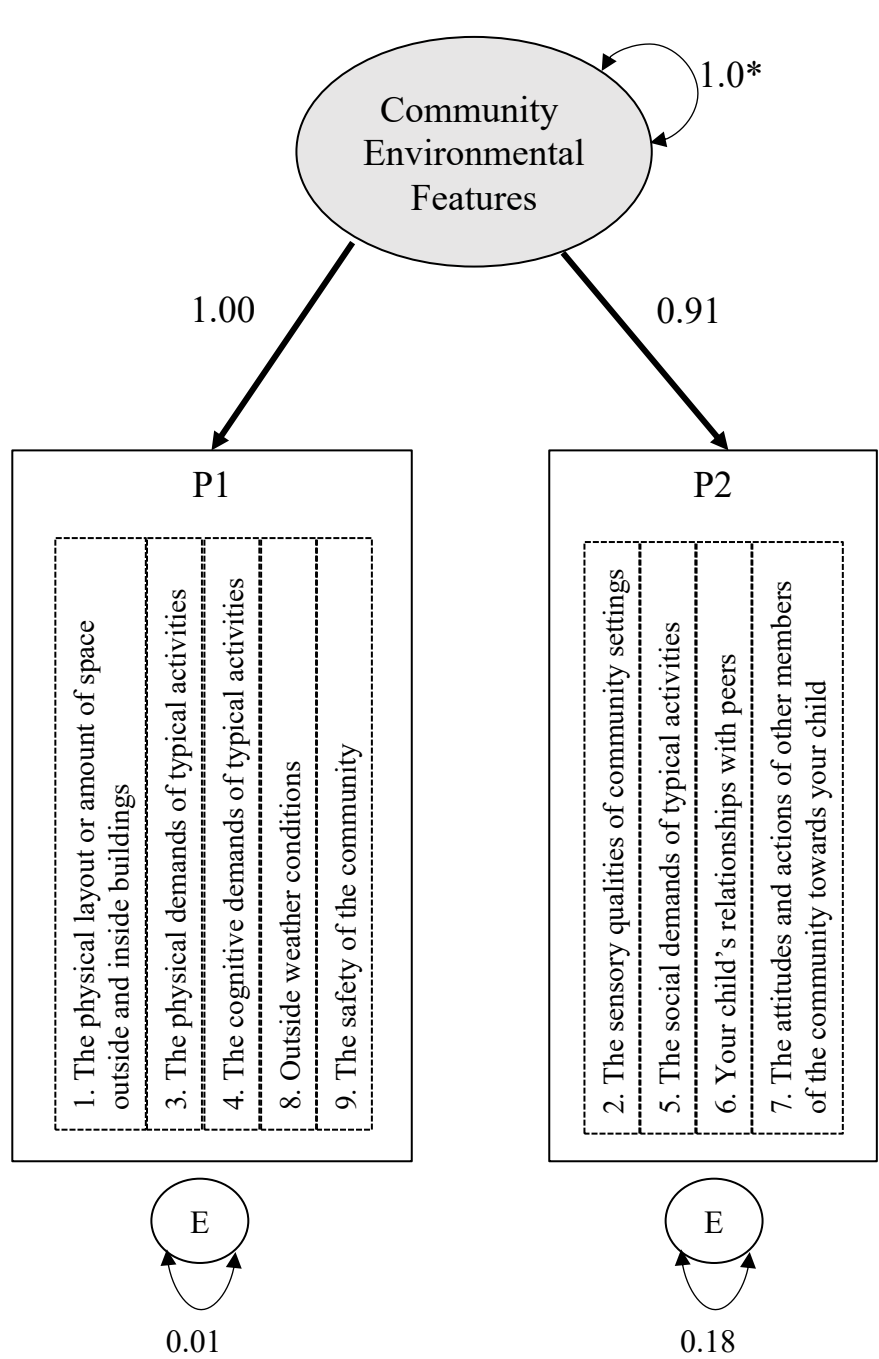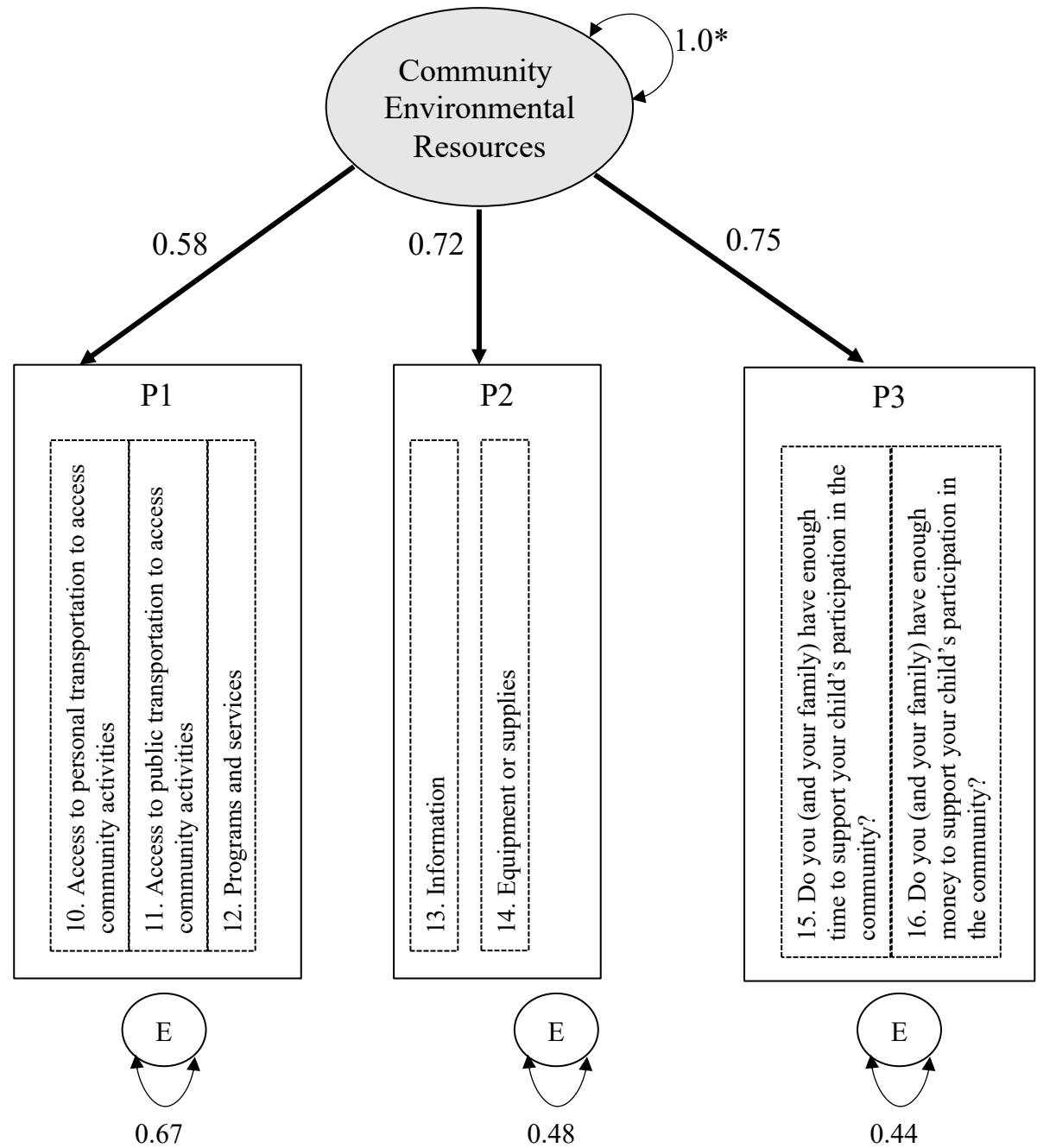

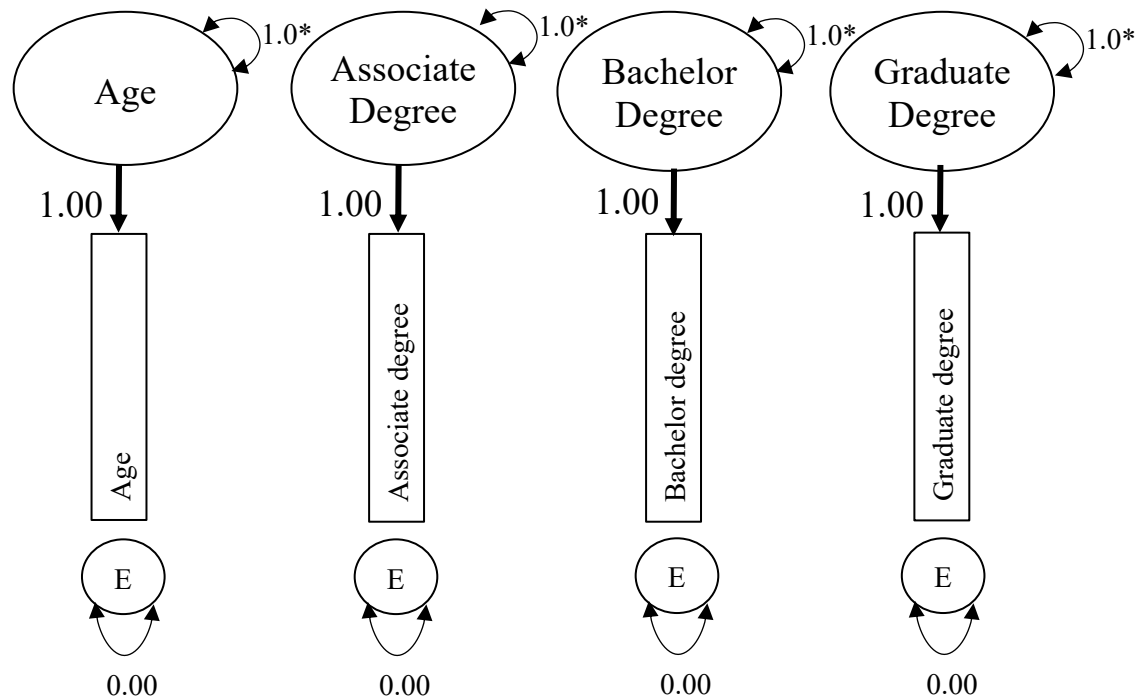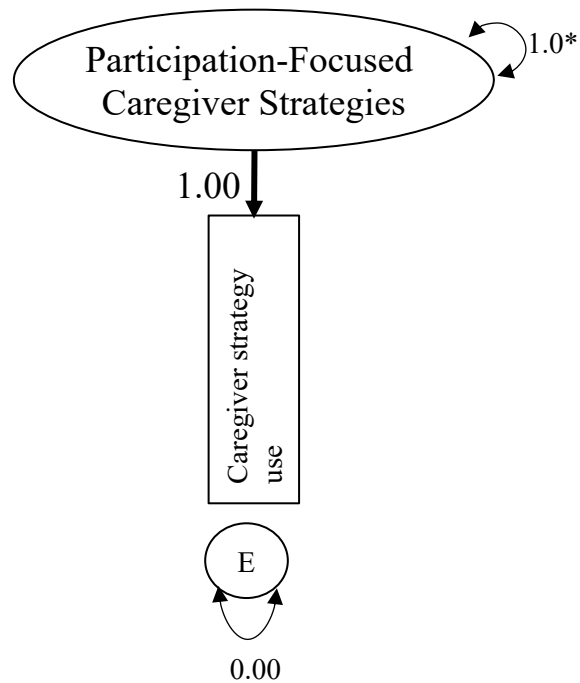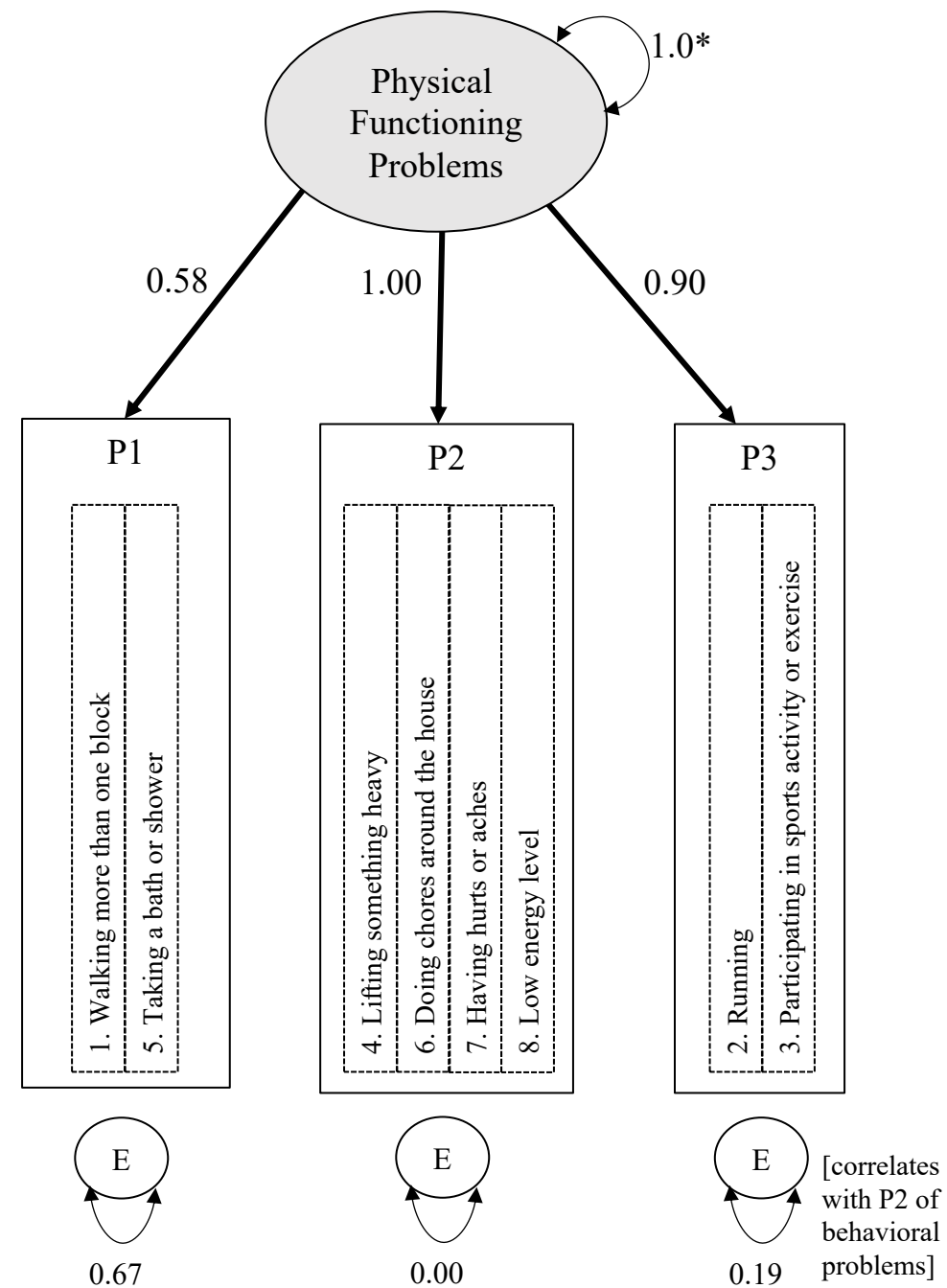

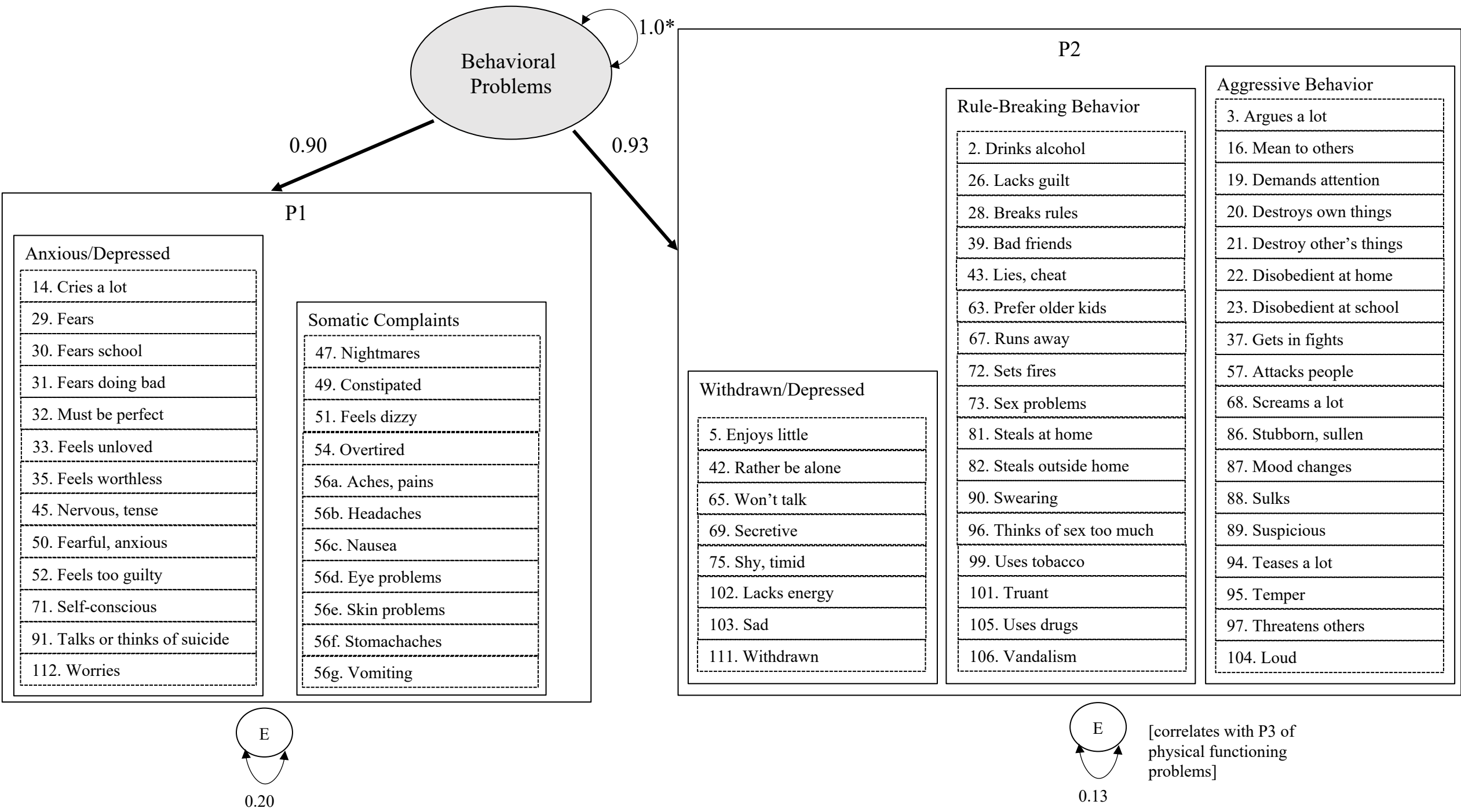

Supplement: Supplementary file 1 [file Image1.pdf]
