# Supplementary material for: Caregiver strategies supporting community participation among children and youth with or at risk for disabilities: a mixed-methods study
Source: Front Pediatr. 2024 Feb 15;12:1345755. doi: 10.3389/fped.2024.1345755 (PMC10902462; doi:10.3389/fped.2024.1345755)
Supplement: Supplementary file 2 [file Datasheet1.pdf]

Interview Guide

Thank you for taking the time to chat with us about your experiences with community participation.

We conducted a project focusing on the use of caregiver strategies to support their child's participation in community activities. In this interview we have some questions to help us better understand our results. So, we really appreciate you sharing your stories.

In this research project we are mainly interested in understanding participation in community activities such as neighborhood outings, community events, and overnight visits or trips.

As outlined in the consent form, this session will be audio and video recorded. Your contributions will be confidential and will not impact your connection with myFace in any way.

Before we begin, we would like to ask you to not share your own or anyone else's name, including your child's name. We would also like to guide you through adjusting your name on Zoom. This is a protocol we are putting in place in order to maintain confidentiality as we move forward with the meeting.

1. Click the button that says 'Participants' on the bottom toolbar
2. Find your name on the pop-up box and click on it
    a. Click 'More' if necessary
3. Click 'Rename'
4. Type in (a number) and then click 'Rename' once more

You're all set!

Do you have any questions for us?

Let's begin.

* Below are example questions. Additional questions were asked based on participants' responses as is consistent with a semi-structured interview format.

<u>Context question (5-10 mins)</u>

1. Without mentioning your child's name, can you give us a general idea about your child – for example what community activities your child likes to do, what your child's personality is like, who your child spends time with?
2. As a caregiver, what does your support system look like?

<u>Questions to help explain the quantitative results</u>

When we use the word 'participation' during this interview we mean attending activities and also being engaged in activities – this includes feeling involved or included when being a part of an activity. Examples for community activities are [show the PEM-CY activities on the powerpoint

slide to give context around community participation AND connect with activities caregiver mentioned in first two questions] We see participation in an activity as something different than doing an activity independently. This means, a child does not have to do the full activity themselves to fully participate. I can be present at an activity and feel included, even though I am not doing the full activity myself. For example, a child could be involved in an organized sports team in their community. Regardless of if the child completes a round on their own or receives help with throwing the ball, they can fully participate.

Let's now look at some of those strategies used in our calculations [show some examples of types of caregiver strategies that were used in the dataset (i.e., community strategies targeting the environment, a child's or youth's sense of self, preferences and activity competencies: 'Try to determine what is happening well ahead of time and plan for it'; 'We also participate in some of the activities'; 'Focus on positive activities my child can do and look at any community calendar or upcoming activities'; 'We always offer words of encouragement and praise'; 'Take favorite snacks to community events'; 'Bring ideas of participation to my child'; 'Guidance'; 'We tell him how he can help others')]
Take a moment to read through the strategies on the screen and pick two strategies and let us know what you think about these strategies.

1. Are these good strategies?
    a. [For good strategies] Why is this strategy a good strategy?
    b. [For bad strategies] Why is this strategy less helpful?

    Prompts:
    c. What factors make this a good/less helpful strategy?
    d. What is missing in this less helpful strategy?
    e. What are you looking for in strategies to decide whether you would try them or not?
    f. What would need to be added to make this a strategy you would try and implement?

2. Are these strategies more or less developed than yours? How did you get to a more developed strategy?

Interestingly, we have found different results for the school setting versus the community setting. Strategies seem to support children and youth in attending school activities, but not in attending or feeling involved in community activities [show powerpoint slide for visual of quantitative results of not having an impact on community participation].

3. What could be reasons for this? [not having an impact in the community setting / to matter in the school setting but not in the community setting]?

    Prompts:
    a. Can you tell me more about […]
    b. Why do you think that?
    c. Do you have any additional thoughts on this that haven't been shared yet?
